# The Effects of Seed Size on Hybrids Formed between Oilseed Rape (*Brassica napus*) and Wild Brown Mustard (*B. juncea*)

**DOI:** 10.1371/journal.pone.0039705

**Published:** 2012-06-22

**Authors:** Yong-bo Liu, Zhi-xi Tang, Henri Darmency, C. Neal Stewart, Kun Di, Wei Wei, Ke-ping Ma

**Affiliations:** 1 State Key Laboratory of Vegetation and Environmental Change, Institute of Botany, Chinese Academy of Sciences, Beijing, China; 2 UMR1347 Agroécologie, Institut National de la Recherche Agronomique, Dijon, France; 3 Department of Plant Sciences, University of Tennessee, Knoxville, Tennessee, United States of America; 4 State Key Laboratory of Environmental Criteria and Risk Assessment, Chinese Research Academy of Environmental Sciences, Beijing, China; 5 PetroChina Tarim Oilfield Company, Korler, Xinjiang, China; Instituto Valenciano De Investigaciones Agrarias, Spain

## Abstract

**Background:**

Seed size has significant implications in ecology, because of its effects on plant fitness. The hybrid seeds that result from crosses between crops and their wild relatives are often small, and the consequences of this have been poorly investigated. Here we report on plant performance of hybrid and its parental transgenic oilseed rape (*Brassica napus*) and wild *B. juncea*, all grown from seeds sorted into three seed-size categories.

**Methodology/Principal Findings:**

Three seed-size categories were sorted by seed diameter for transgenic *B. napus*, wild *B. juncea* and their transgenic and non-transgenic hybrids. The seeds were sown in a field at various plant densities. Globally, small-seeded plants had delayed flowering, lower biomass, fewer flowers and seeds, and a lower thousand-seed weight. The seed-size effect varied among plant types but was not affected by plant density. There was no negative effect of seed size in hybrids, but it was correlated with reduced growth for both parents.

**Conclusions:**

Our results imply that the risk of further gene flow would probably not be mitigated by the small size of transgenic hybrid seeds. No fitness cost was detected to be associated with the *Bt*-transgene in this study.

## Introduction

Transgene flow from crops to their wild relatives is a potential risk associated with commercial release of transgenic crops [1]. A review by Ellstrand *et al.* notes that 12 out of the world's 13 most important crops could hybridize with wild relatives. This suggests that transgenes could escape via spontaneous hybridization with and introgression to wild relatives [2], though the risk has to be assessed according to crop characteristics and mitigation strategies [3]. Escape of transgenes into wild populations could potentially enhance the invasiveness of weeds, because transgenes provide adaptive traits such as resistance to pests, herbicides, viruses and other diseases, and various environmental stresses [4]. Hybrid transgenic progeny have been observed both within and outside of agro-ecosystems, such as in fields, their margins and roadsides [5], [6].

The fate of hybrids will have a crucial effect on gene flow and the potential for subsequent introgression of transgenes into wild and weedy hosts. Seed formation may be the first important phase of hybrid development, because parental differences in chromosome numbers and certain traits could negatively affect embryo development. For instance, studies of interspecific hybridization between oilseed rape (OSR, *Brassica napus*) as female and its wild relatives as pollen donors have shown that hybrid seeds are all small relative to either parent when hybridizing with *Hirschfeldia incana* [7], [8], *Raphanus raphanistrum* [7], [8] and *B. juncea* [9] but partially small with *B. rapa* [10]. Wei and Darmency later confirmed that all hybrid seeds produced by a male-sterile OSR cultivar and four wild species were within the smallest size class, except the hybrids formed with *B. rapa*, for which hybrids seeds were equally distributed among all seed-size classes [11]. The small size of hybrid seeds that being smaller than either parent could explain the absence of recorded hybrids–other than *B. napus*×*B. rapa* hybrids [11]–because small seeds purportedly confer a fitness disadvantage due to negative effects on emergence, initial seedling size, and early competitive ability [12], [13]. Decreased fitness in small-seeded plants could then be exacerbated under stressful conditions such as high density, shade, drought, or herbivory [14], [15]. Thus, hypothetically, gene flow could be hampered by small sized seeds in hybrids between transgenic crops and wild relatives [11]. However, the negative effect of hybrid seed size was only observed in the field at the early stage of seedling establishment, and plant growth was evaluated without accounting for seed production and competition among plants of different seed sizes. Since seed size is an important factor in ecology [16] that can affect the process and consequences of gene flow, we investigate here whether plants grown from small seeds do indeed have a reduced competitive ability compared to plants grown from larger seeds.

Oilseed rape is considered to be a crop at high risk for introgression into wild populations [3]. *B. juncea*, an allotetraploid wild relative species, is frequently found as a weed and a ruderal component of roadsides and waste places in China. Open pollination experiments have successfully generated hybrids and subsequent backcross generations between *B. napus* and wild *B. juncea* [9], [17], [18]. Transgene introgression is a biosafety concern because of the relatively high compatibility between *B. napus* and wild *B. juncea*. Therefore, we wonder whether *B. juncea*×*B. napus* crosses actually produce small hybrid seeds, comparing to their parents, and, if so, does small seed size impact the fate of hybrids compared to either volunteer and feral *B. napus* or wild *B. juncea*, and thus reduces gene flow in nature? In this study, we focus on seed-size components of plant growth and reproduction in the agro-ecosystem and on the interaction between seed size and transgene introgression. The objectives of this study were to investigate 1) whether there is any effect of seed size on plant growth and reproduction; if so, 2) whether the effects vary with plant type and plant density and 3) whether the exotic gene (transgene) modifies these effects; and to discuss 4) whether small-seeded hybrids impede further gene flow, considering seed survival in the field and potential change of both fertility and seed size in further generation. In our study, the hybrid seeds have been produced using wild plants as female parent, which could indicate what might happen in the wild (outside the arable fields), as well as in weedy populations (within the fields, in competition against oilseed rape). In contrast to most documented cases, in which seed size is expected to be intermediate between the two parents, the hybrids seeds here are smaller than those of either parent; plant performance and seed production of hybrids in the field could be outside the parental range. The experiments were performed under various conditions of plant competition at different levels of plant density.

## Materials and Methods

### Plants

Seeds of wild brown mustard (*Brassica juncea*, 2n = 36, AABB), sampled from a local field collection, were provided by Nanjing Agricultural University, China. *Brassica napus* cv. “Westar” (OSR, 2n = 38, AACC), a spring-type OSR, was transformed with the pSAM 12 plasmid containing genetically linked *GFP* and *Bt Cry1Ac* cassettes regulated by independent CaMV 35S promoters [21]. The third generation of transgenic plants generated from the homogenous T_2_ transgenic event 1 (GT_1_) was bulked in the Institute of Botany, Chinese Academy of Sciences (IBCAS) to obtain the fourth generation, which allowed sufficient seeds and plants with stable expression of *Bt* and *GFP* genes. Transgenic F_1_ hybrids (trF_1_) were formed between wild mustard (as female) and the fourth generation of GT_1_. Non-transgenic F_1_ hybrids (ntrF_1_) were obtained by crossing wild mustard (used as the female parent) with the non-transgenic OSR parent (Westar). Both hybridization experiments used ten paternal and maternal plants.

The GT seeds were sieved into three categories by seed diameter: larger than 1.6 mm (L), smaller than 1.2 mm (S), and intermediate between 1.2 and 1.6 mm (M). These categories were the same as those used in previous studies on the hybridization of *B. napus* with five wild relatives [11] and resulted in balanced classes around the M category, which contained nearly half the seeds (Table 1). Seeds of wild mustard were smaller, thus 1.2 mm and 1.0 mm sieves were used for the seed-size classification, which again resulted in balanced classes around the main M category (Table 1). Most hybrid trF_1_ and ntrF_1_ seeds were smaller than 1.2 mm and were sorted into four size categories. However, the smallest seeds had little-to-no germination in preliminary research [19], so the distribution of the seeds used in this study was shifted upwards in category, and sieved by 0.9 and 1.0 mm (Table 1). Plants derived from the three seed-size categories were subsequently labelled as small-seeded plants (SSP), medium-seeded plants (MSP) and large-seeded plants (LSP) in the following experiments.

**Table 1:** Distribution of seed-size categories for the four plant types (modified from reference [19]).

Plant types	Seed size (mm)	Percentage
GT	S (<1.2)	26.35
	M (1.2–1.6)	44.86
	L (>1.6)	28.79
trF1	<0.8	22.05*
	S (0.8–0.9)	35.65
	M (0.9–1.0)	22.81
	L (>1.0)	19.49
ntrF1	<0.8	32.09*
	S (0.8<0.9)	30.72
	M (0.9–1.0)	16.83
	L (>1.0)	20.36
Mustard	S (<1.0)	34.96
	M (1.0–1.2)	52.65
	L (>1.2)	12.39

* Not used because less than 4% germination.

### Monoculture experiment

The three seed categories of *B. juncea* (mustard) were sown in the experimental field of the botanical garden of IBCAS on March 19, 2008. The experimental design consisted of three blocks, each having three plots with a different plant density: 400 plants/m^2^ (high), 100 plants/m^2^ (medium) and 25 plants/m^2^ (low) (Fig. S1). Each plot had 60 sowing positions at the intersections of 5 rows and 12 columns arranged in three distances: 5 cm, 10 cm and 20 cm, for the high, medium and low densities, respectively. Ten centre positions and ten edge positions in each plot were randomly assigned to each seed category and each position was sown with five seeds. So, for example, small seeds of mustard were sown in 10 randomly-selected positions in the center of each plot and in 10 randomly-selected positions on the edge. Emerged seedlings were counted, and then randomly thinned to one plant per position.

### Multi-culture experiment

In order to study interspecific competition, the four types of plants–mustard, GT, trF_1_, and ntrF_1_–were co-cultivated together. Seeds from each of the three size categories were directly sown in the field on March 31, 2008. There were three blocks in this experiment and each block contained two plots at two plant densities: 100 plants/m^2^ and 25 plants/m^2^, i.e. the distance between plants at these two densities was 10 cm and 20 cm, respectively (Fig. S1). Five seeds of each seed category for each plant type were sown at 10 randomly-selected positions in each plot. In total, there were 120 positions for three seed categories of four plant types, sown in 10 rows and 12 columns per plot. The number of emerged seedlings were recorded, and then randomly thinned to one plant per position, as in the monoculture trial above.

### Measurements

These two experiments were kept weed-free by hand weeding, and every plant was labelled. Field management of the plants was identical to normal OSR cultivation, and included five pesticide treatments applied during the growth season. The weather was within normal regional ranges during the experiments. Open pollination was permitted during the flowering period.

The following variables were measured for every plant in both experiments (unless otherwise noted): the number of days from sowing to flowering; number of flowers (in the monoculture trial only), seed number, seed weight, and total dried aboveground biomass. Thousand-seed weight was calculated for each plant. Reproductive allocation was estimated as seed production per unit biomass (seed weight/biomass). The harvested seeds produced by each plant were sieved into three categories by seed diameter, as described above, and the number and total weight of seeds in each seed category were measured. The percentage of seeds in each of the three seed categories set on each individual plant was calculated.

### Statistical analysis

In order to prevent border effects, only plants in plot centres were included (i.e.10 plants per category for monoculture, and a mean number of 6.7 per category for the multi-culture experiment), and averages per block were used for statistical analyses. A three-way fixed split-plot ANOVA model was used for the monoculture experiment, which included plant density (D) as the main plot tested against the density:block interaction (D:B), seed size (S), block (B) and density:seed size interaction (D:S) (Y∼D* S+B+D:B in R language). A four-way fixed split-plot ANOVA model (Y∼D* S* P+B+D:B) was used for the multi-culture experiment, which included density as main plot, seed size, block and plant type effects (P), D:S, D:P, S:P and D:S:P interactions. In these two models, the mean square of D:B was used as the error of D to calculate its F-value, because the density was split into plots in each block while other factors were randomly distributed within each plot. All data were log-transformed to improve the normality of errors and homogeneity of variance. The split-plot ANOVA results, including the main effect of each factor and their interaction, are provided in supplementary Tables S1 and S2. When there were significant interactions among factors (D, S, P), the related variables were analysed using Bonferroni corrected pair-wise comparisons and Tukey's honestly significant difference (Tukey's HSD) test. All statistical analyses were conducted in R software version 2.13.1 [20].

## Results

### Monoculture experiment

**Figure 1:**
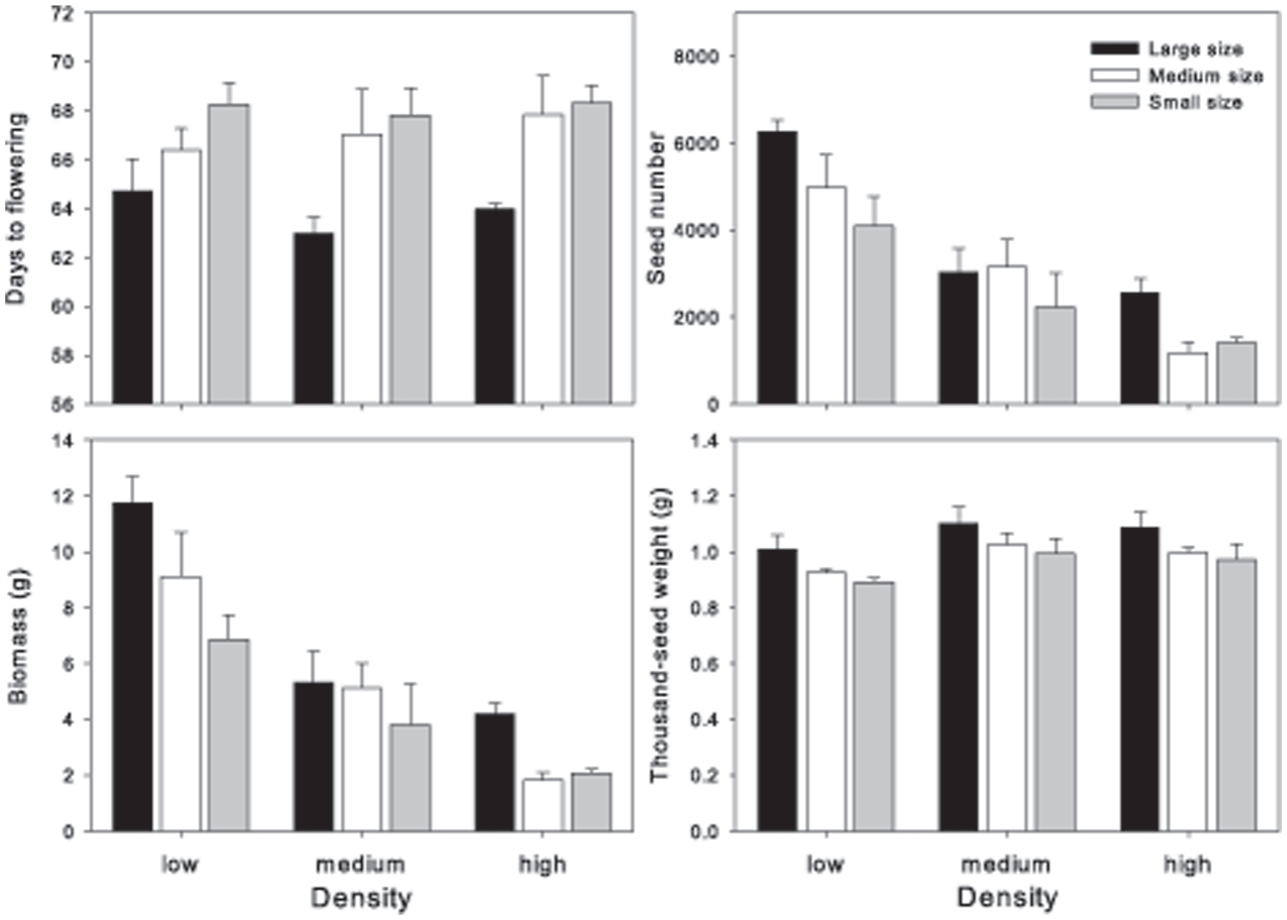
Four plant growth characteristics of the three seed-size categories of wild *Brassica juncea* at three plant densities in the monoculture experiment. Each group of bars represents plants derived from three seed-size categories for a given plant density. Significant effects of seed size were observed for the four traits. Effects of density were observed for flowering date and seed number only.

**Effects of plant density**: Plant density significantly affected the performance of mustard plants; increased density decreased the number of flowers, biomass, and seed number and weight, and increased the percentage of large and medium seeds (Tables 2 and Table S1, Fig. 1). Flowering time, reproductive allocation and thousand-seed weight were not affected by plant density.

**Table 2:** Means (±SE) of growth characteristics of the mustard plants in the monoculture experiment at three densities and for plants derived from three seed-size categories: LSP, MSP and SSP for large-, medium- and small-seeded plants, respectively.

	Density			Seed size		
	Low	Medium	High	LSP	MSP	SSP
Emergence rate	0.62(0.05)	0.58(0.04)	0.57(0.05)	0.62(0.05)	0.58(0.05)	0.56(0.05)
Days to flowering	66.4(0.7)	65.9(1.0)	66.7(0.8)	63.9(0.5)^b^	67.1(0.8)^a^	68.1(0.5)^a^
No. of flowers	38.9(5.5)^a^	21.9(3.3)^b^	13.3(1.7)^c^	33.2(6.1)^a^	23.3(4.3)^b^	17.5(3.7)^b^
Biomass	9.23(0.92)^a^	4.75(0.63)^b^	2.69(0.40)^c^	7.09(1.25)^a^	5.35(1.18)^b^	4.24(0.86)^b^
Seed number	5112(437)^a^	2801(365)^b^	1708(247)^c^	3952(616)^a^	3098(622)^b^	2571(501)^b^
Seed weight	5.10(0.54)^a^	3.10(0.44)^b^	1.86(0.32)^c^	4.43(0.62)^a^	3.19(0.65)^b^	2.44(0.43) ^a^
Reproductive allocation	0.54(0.02)	0.60(0.02)	0.58(0.03)	0.60(0.03)	0.54(0.02)	0.57(0.02)
Thousand-seed weight	0.94(0.02)	1.04(0.03)	1.02(0.03)	1.07(0.03)^a^	0.98(0.02)^b^	0.95(0.02)^b^
Percentage of small seeds	0.75(0.04)^b^	0.51(0.05)^a^	0.49(0.04)^a^	0.49(0.03)^b^	0.62(0.03)^a^	0.64(0.03)^a^

Different letters within a row for both density and seed size indicate significantly different values at P<0.05.

**Effects of seed size**: Seed-size category affected all traits except the emergence rate and the reproductive allocation (Tables 2 and Table S1, Fig. 1). LSP flowered earlier than SSP by an average of 4 days, and produced 89% more flowers, 53% more biomass, 41% more seeds, 70% higher seed weight and had more large seeds but fewer small seeds. Thousand-seed weight in LSP was 13% greater than that in SSP (Table 2 and Table S1). There was no interaction between density and seed size for emergence rate, biomass, seed number and weight (Table S1). In all seed-size classes, there was a strong positive correlation between number of flowers, biomass, seed number and seed weight (P<0.001), and the percentage of small seeds was negatively correlated with the percentage of large seeds (P<0.001). Thousand-seed weight was positively correlated (P<0.01) with these same four traits–number of flowers, biomass, seed number and seed weight–for LSP and MSP, but there was no correlation for SSP.

### Multi-culture experiment

**Effect of plant density and seed size**: Increased plant density reduced biomass, and seed number and weight. The other traits were not affected (Tables 3 and Table S2). The significant effect on the emergence rate was due to important block and main plot bias; GT values were 0.35 at the low density and 0.68 at the high density. This bias had no influence on subsequent measurements, because only one seedling was left at each position. SSP had significantly delayed flowering (by two days), lower biomass, lower seed number and weight, and lower thousand-seed weight than LSP (Table 3 and Table S2). There was no interaction between density and seed size for any measured characteristics (Table S2), indicating that seed-size effects persisted at all densities. Interactions between density and plant type and between seed size and plant type were observed, and are described below. There were no interactions between plant density, seed size, or plant type and any of the measured plant growth characteristics (Table S2).

**Table 3:** Means (±SE) of growth characteristics of plants in the multi-culture experiment at two densities and for plants derived from three seed-size categories: LSP, MSP and SSP for large, medium and small-seeded plants, respectively.

	Density		Seed size		
	Low	High	LSP	MSP	SSP
Days to flowering	57.3(0.6)	58.7(0.8)	56.9(0.9)^a^	58.2(0.9)^a^	58.9(0.8)^a^
Biomass	18.19(1.72)^a^	9.69(1.35)^b^	17.53(2.33)^a^	11.71(1.59)^b^	12.56(2.13)^b^
Seed number	1406(240)^a^	565(101)^b^	1351(306)^a^	810(186)^b^	796(203)^b^
Seed weight	2.91(0.39)^a^	1.05(0.27)^b^	2.22(0.60)^a^	1.22(0.28) ^b^	1.01(0.25)^ b^
Reproductive allocation	0.19(0.04)	0.14(0.03)	0.17(0.04)	0.20(0.07)	0.13(0.03)
Thousand-seed weight	1.20(0.08)	1.31(0.12)	1.31(0.09)^a^	1.31(0.11)^a^	1.15(0.08)^b^
Percentage of small seeds	0.34(0.06)	0.34(0.06)	0.32(0.07)	0.31(0.07)	0.37(0.07)

Different letters within a row for both density and seed size indicate significantly different values at P<0.05.

**Figure 2:**
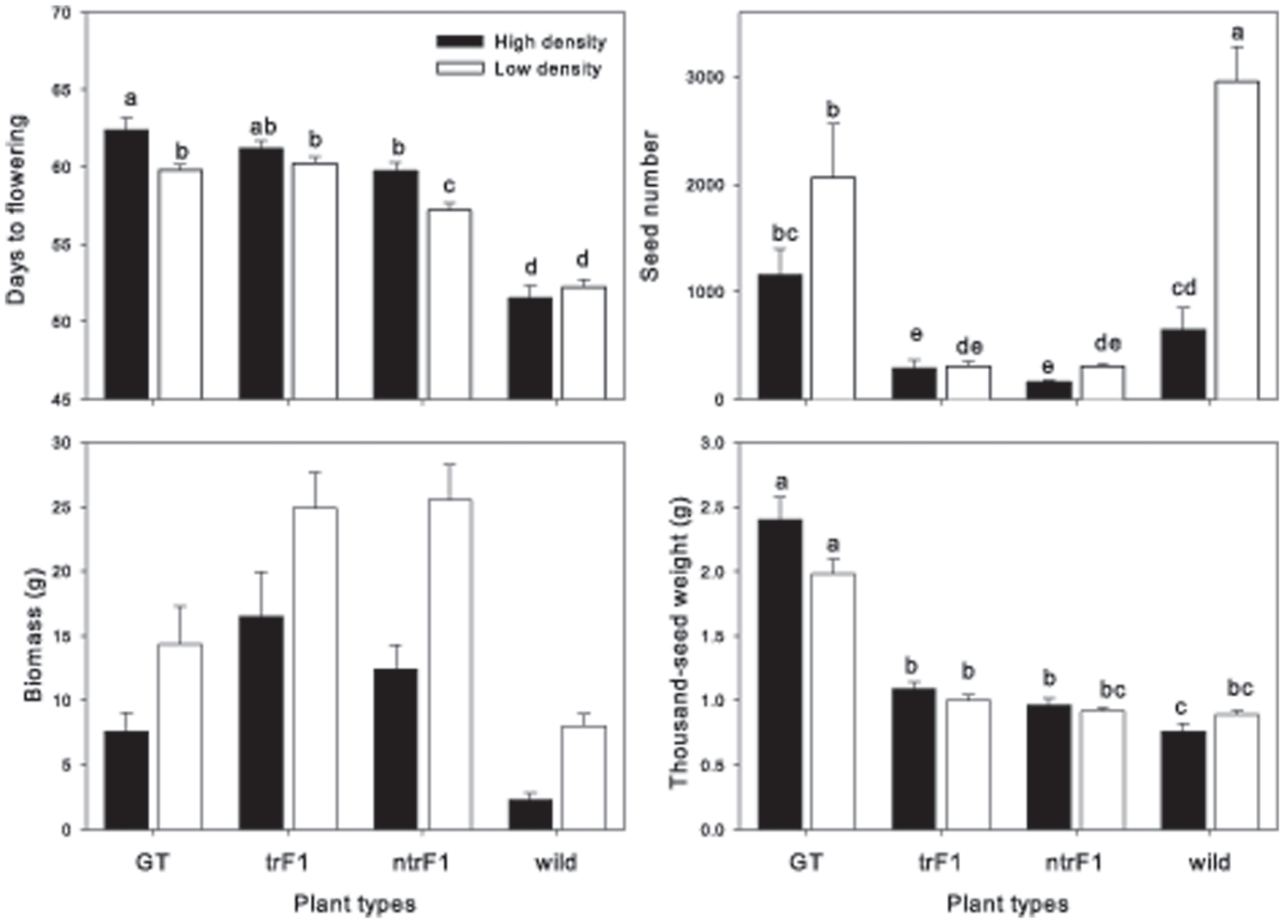
Four plant growth characteristics of the four plant types–transgenic *B. napus* (GT), transgenic F1 (trF1), non-transgenic F1 (ntrF1) and wild *B. juncea* (wild)–at high density (black bar) and low density (white bar) in the multi-culture experiment. Different letters indicate significantly different values, evaluated using Tukey's HSD test at P<0.05.

**Figure 3:**
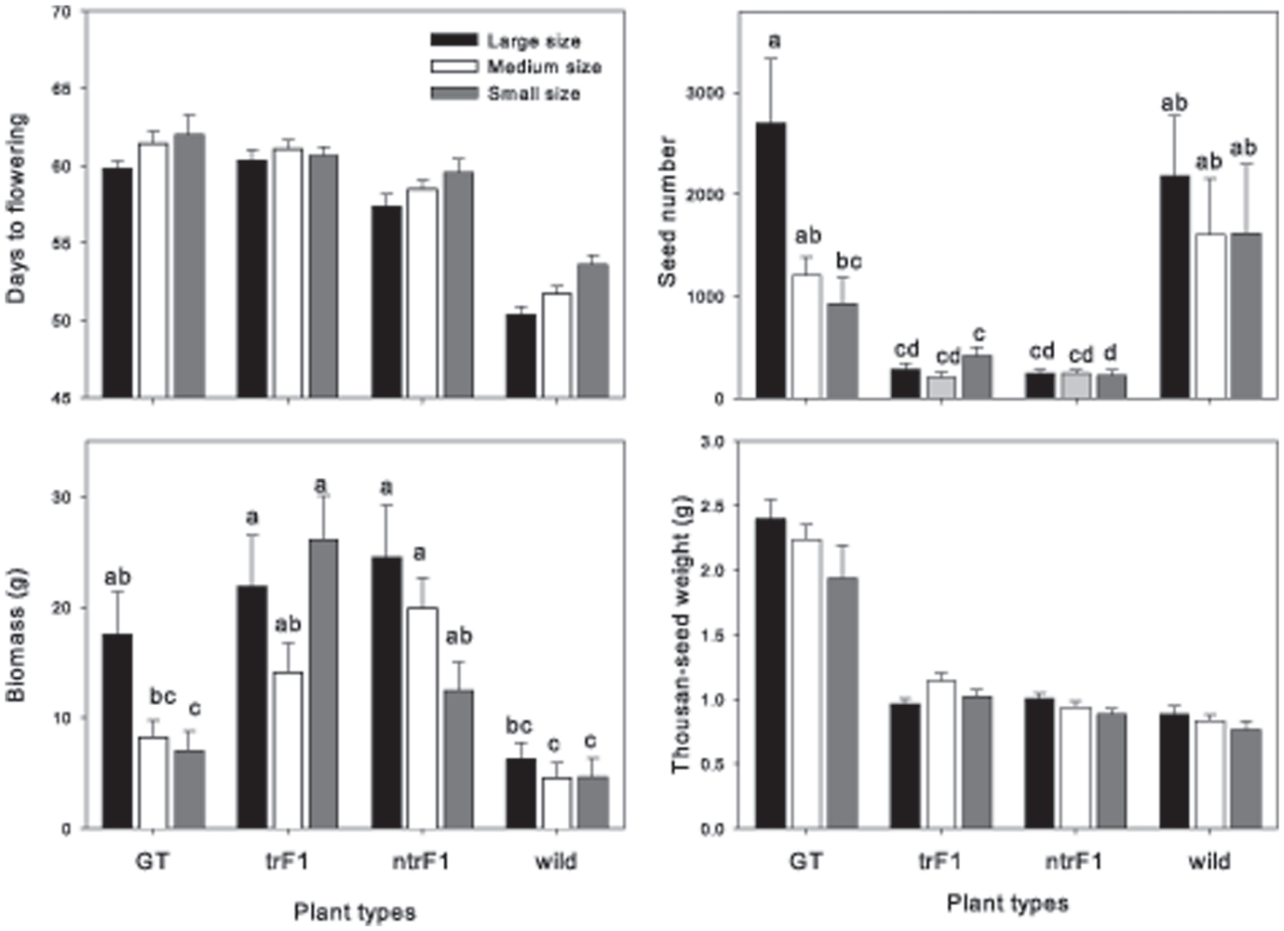
Four plant growth characteristics of the four plant types–transgenic *B. napus* (GT), transgenic F1 (trF1), non-transgenic F1 (ntrF1) and wild *B. juncea* (wild)–for three seed categories (large, medium and small in black, white and grey, respectively) in the multi-culture experiment. Significant effects were observed for seed number and biomass only. Different letters indicate significantly different values, evaluated using Tukey's HSD test at P<0.05.

**Performance of the four plant types**: All measured growth characteristics differed among the four plant types: GT, trF_1_, ntrF_1_ and mustard (Tables 4 and Table S2, Fig. 2 and 3). Wild mustard flowered earliest, then ntrF_1_ and finally trF_1_ and GT. We observed transgressive segregation, as the hybrids (trF_1_ and ntrF_1_) produced greater biomass, fewer seeds, and lower seed weight and reproductive allocation than both parents. As expected, mustard plants had the lowest biomass and greatest reproductive allocation, and GT plants had the highest seed number and seed weight (Table 3). The thousand-seed weight of both hybrids, trF_1_ and ntrF_1_, was intermediate to that of the wild and GT parents. The distribution of the three seed-size categories in harvested seeds shifted toward larger seeds for the GT parent (53%) and the trF_1_ and ntrF_1_ hybrids (78 and 73%), while it shifted toward smaller seeds for the wild parent (91%) (Table 4). We also found differences between the two hybrids: trF_1_ plants flowered later and had higher seed weight and greater reproductive allocation than the ntrF_1_ plants (Table 3). Traits were correlated in the same way as in the monoculture experiment, e.g. seed number and seed weight were positively correlated (P<0.001), except that thousand-seed weight was not correlated to biomass, seed number and weight in GT, trF1 and ntrF1. Thousand-seed weight was significantly correlated with these three variables (at least P<0.02) for mustard grown from all three initial seed categories.

**Table 4:** Means (±SE) of plant growth characteristics for the four plant types used in the multi-culture experiment.

	GT	trF1	ntrF1	mustard
Days to flowering	61.1(0.5)^a^	60.7 (0.3)^a^	58.5 (0.5)^b^	51.9(0.4)^c^
Biomass (g)	10.9 (1.82)^b^	20.69 (2.40)^a^	18.96 (2.24)^a^	5.15 (0.85)^c^
Seed number	1608(290)^a^	298 (43)^b^	233(25)^b^	1802(334)^a^
Seed weight (g)	3.64 (0.64)^a^	0.35 (0.07)^c^	0.22 (0.02)^d^	1.71 (0.34)^b^
Reproductive allocation	0.285 (0.025)^a^	0.016 (0.004)^b^	0.014 (0.003)^c^	0.346(0.079)^a^
Thousand-seed weight (g)	2.19 (0.11)^a^	1.04 (0.03)^b^	0.94 (0.03)^b^	0.83 (0.04)^c^
Percentage of small seeds	0.16 (0.02)^b^	0.13 (0.01)^b^	0.18 (0.02)^b^	0.91 (0.01)^a^

trF1: transgenic hybrids formed between transgenic oilseed rape (GT) and *Brassica juncea* (mustard); ntrF1: non-transgenic hybrids formed between non-transgenic oilseed rape “Westar” and *B. juncea* (mustard); mustard: wild mustard (*B. juncea*); GT: transgenic oilseed rape. Different letters within a row indicate significantly different values at P<0.05.

**Interactions among plant density, plant type and seed size**: GT and hybrids flowered later in the high density plots than in low density plots, in contrast to the mustard, which was not affected by density (Fig. 2, Table S2). Seed number and seed weight of mustard were greatly reduced at the high density, but not for the other plant types (Fig. 2). At the high density, thousand-seed weight was slightly reduced for mustard, increased for the OSR GT, and unaffected in the hybrids; however, this effect was weak after Bonferroni adjustment (Fig. 2). Finally, SSP GT parents had markedly lower biomass and seed production than LSP GT parents, but there was no significant difference in these variables for different seed sizes of hybrids or mustard (Fig. 3).

## Discussion

In the *B. juncea* monoculture experiment, the flowering time, biomass, number of flowers and seeds, and thousand-seed weight of wild mustard were affected by the seed-size category from which the plants were grown. A similar result was observed in the multi-culture experiment for all four plant types, which suggests that seed size is an important determinant of plant fitness. This is consistent with findings in other studies. For instance, in *R. raphanistrum*, larger seeds generally produce larger plants with more flowers than those that originated from smaller seeds [22], [23]. The earlier flowering seen in larger seeded plants in both of our experiments might have resulted from earlier emergence, as noted by Harper [24], although we did not record emergence date in our study. It is generally thought that seed mass has a significant influence on seedling emergence and initial plant development both within species or varieties [11], [12], [25], [26] and among species [13], [27]. After the early seedling stage, plant growth and development rates are similar regardless of seed size, as the principal energy sources become photosynthates from emerged leaves [12], [23], [28]; this means that initial differences related to seed size could be compensated at later growth stage. Indeed, reproductive allocation was not significantly differentiated among all densities and seed sizes in this study.

In our multi-culture experiment, the seed-size effect was mainly driven by the *B. napus* response and plants originated from smaller seeds tended to have decreased seed production. Though it followed the same trend as in the monoculture experiment, the initial seed-size effect was not significant for *B. juncea* in the multi-culture experiment. A previous study [11] suggested that seed-size effects were predominant at the early stage of seedling establishment, with very little seed-size effect afterward. In our study, for the most part, plants were thinned to leave only the biggest, healthiest seedling per position, thus hiding part of the early differential effect of seed size. Although it is often the case that small-seeded plants were more vulnerable to environmental stress than larger-seeded plants [29], seed-size effects on plant fitness could be balanced by plant types and environmental stresses, such as high plant density and herbivory [12], [23], [28]. For instance, a seed-size effect was observed in a direct sowing experiment but not in a transplant experiment [11]. The authors suggested that the homogeneous environment in the greenhouse prior to transplantation into the field had compensated for the small seed disadvantage at the early growth stage. Another study reported an interaction of plant density on the influence of seed size on plant growth and reproduction [14]. In our study, the lack of density*seed size interaction in both experiments showed that the performance of small-seeded plants was not density-dependent within the 25 to 400 plants/m^−2^ range of our experiments–a density range that is quite representative of plant densities occurring in oilseed rape fields (including the crop and any other weeds). However, it is not possible to extrapolate this result to roadsides and waste places where established vegetation could affect seedling emergence and growth, especially for small seeded plants.

Seed size significantly affected the performances of transgenic oilseed rape, but it did not impact any growth components measured in the transgenic F_1_ plants. Although the average seed size of hybrids was markedly smaller than that of either parent, they produced a significantly greater proportion of large seeds (diameter>1.0 mm). Therefore, even if seed size had an actual impact on fitness, this negative effect might disappear in further generations.

Hybrids had dramatically decreased fertility– seven-fold less than their parents–as already shown in most *B. napus* hybridization studies [5], [6], [7], [8], [9], [10], [11], but have produced more biomass than either parent, potentially owing to their reduced relative reproductive allocation. However, the fertility could be recovered in subsequent generations [1], [4], [17]. This suggested that hybrid plants could only have disadvantage at earlier steps of introgression.

Small seeds are more easily sieved out by mechanical harvesters and dispersed through wind and animals than large seeds; large seeds would likely be collected together with the crop seeds at harvest. Survival of small seeds could be higher than that of larger seeds in conventional tillage systems and arable habitats, thus creating a potential persistence problem [30]. Hence, small-size seeds produced by hybridization might represent one crucial risk of transgene escape in the field.

We compared two hybrid types in the multi-culture experiments. Performance of small-seeded transgenic F_1_ hybrids was equivalent to that of plants from large seeds. There were no significant differences between transgenic F_1_ and non-transgenic F_1_ plants in seed output, which indicates an absence of transgene-associated fitness costs [31]. A fitness cost of transgene expression has been associated with herbivore resistance in some studies [32], [33], but this cost has not been observed in transgenic *B. napus* and sunflower [31], [34], [35]. The greater overall seed weight and reproductive allocation that we observed in transgenic F_1_ plants were due to the slight increase in both seed number and thousand-seed weight, which suggests that development may have been enhanced in transgenic hybrids. *Bt* resistance might have provided better plant protection against insects during the time periods between pesticide treatments. Certainly, the relative performance of the two hybrids and their wild parent would have shifted under high insect pressure, as observed elsewhere [36], [37], which could suggest that the benefit due to the transgene is likely to be much more important than any possible seed-size effect. If this is true, small seed size could not reduce the risk of gene flow from transgenic crops to wild relatives.

## Supporting Information

Figure S1 **Experimental designs, showing one representative block of monoculture (A) and multi-culture (B) experiments.** Different sizes of plots indicate different plant densities (main plots). Three seed categories and plant types were sown randomly in 60 (A) and 120 (B) plant positions in each main plot.
(TIF)

Table S1 **F-values from a split-plot ANOVA on the plant growth characteristics of wild mustard in the monoculture experiment.**
(DOC)

Table S2 **F-values from a split-plot ANOVA on the plant growth characteristics of transgenic hybrid, non- transgenic hybrid, wild mustard and transgenic oilseed rape in the multi-culture experiment.**
(DOC)
