# Supplementary figures and images for: The Effects of Seed Size on Hybrids Formed between Oilseed Rape (Brassica napus) and Wild Brown Mustard (B. juncea)
Source: PLoS One. 2012 Jun 22;7(6):e39705. doi: 10.1371/journal.pone.0039705 (PMC3382164; doi:10.1371/journal.pone.0039705)

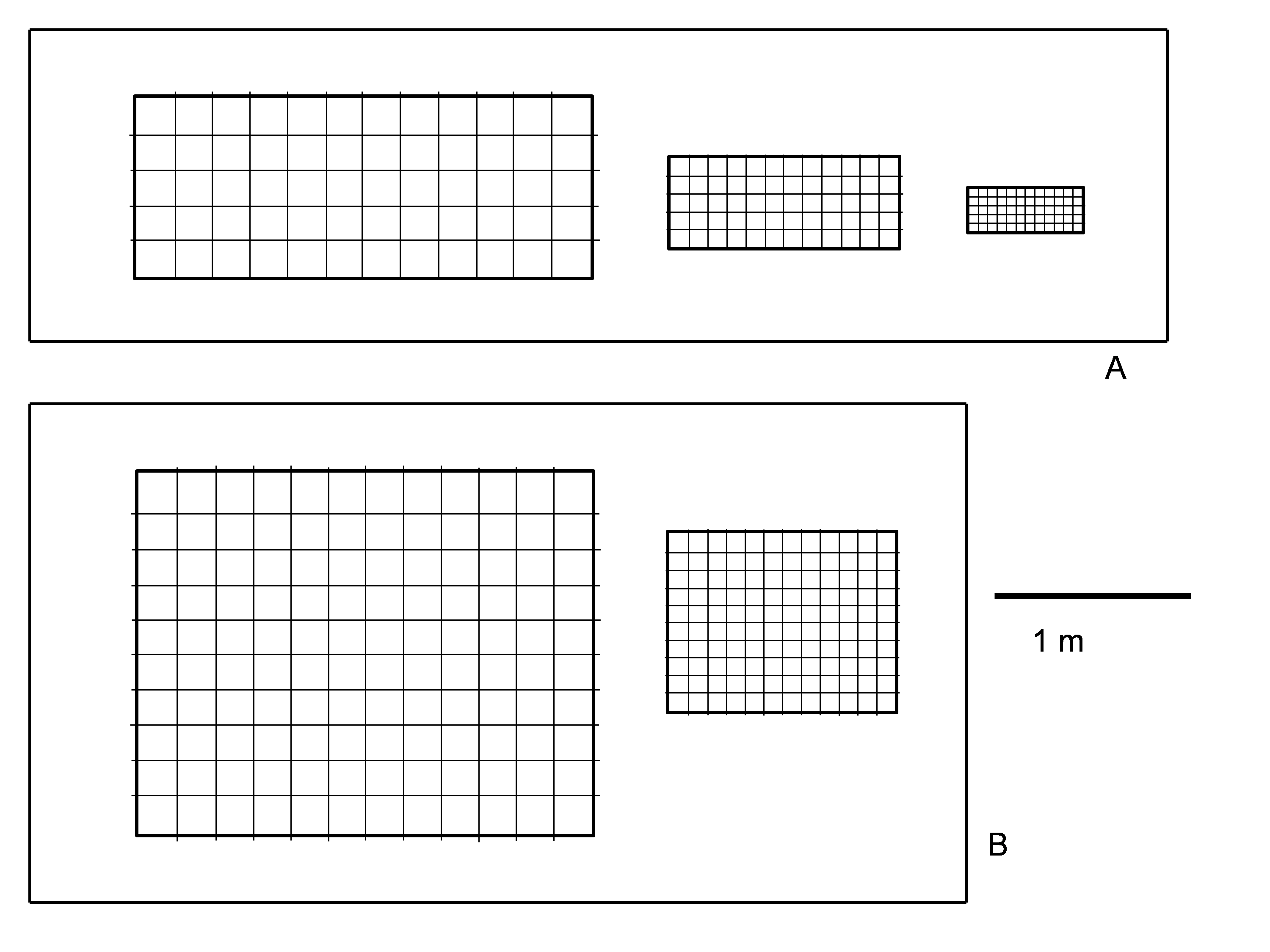

Supplement: Figure S1 — Experimental designs, showing one representative block of monoculture (A) and multi-culture (B) experiments. Different sizes of plots indicate different plant densities (main plots). Three seed categories and plant types were sown randomly in 60 (A) and 120 (B) plant positions in each main plot. (TIF) [file pone.0039705.s001.tif]
