# Supplementary material for: The Effects of Seed Size on Hybrids Formed between Oilseed Rape (Brassica napus) and Wild Brown Mustard (B. juncea)
Source: PLoS One. 2012 Jun 22;7(6):e39705. doi: 10.1371/journal.pone.0039705 (PMC3382164; doi:10.1371/journal.pone.0039705)
Supplement: Table S1 — F-values from a split-plot ANOVA on the plant growth characteristics of wild mustard in the monoculture experiment. (DOC) [file pone.0039705.s002.doc]

Table S1. F-values from a split-plot ANOVA on the plant growth characteristics of wild mustard in the monoculture experiment

|  | df | Emergence rate | Days to flowering | No. of flowers | Biomass | Seed number | Seed weight | Reprod. allocation | Thousand-seed weight | % of large seeds | % of medium seeds | % of small seeds |
| --- | --- | --- | --- | --- | --- | --- | --- | --- | --- | --- | --- | --- |
| Block | 2 | 3.89^*^ | 2.05 | 1.08 | 0.45 | 0.92 | 1.04 | 4.33^*^ | 1.39 | 1.64 | 0.33 | 1.50 |
| Density | 2 | 0.23 | 0.31 | 8.56^*^ | 54.99^**^ | 69.02^***^ | 48.15^**^ | 2.01 | 2.38 | 11.01^*^ | 11.22^*^ | 4.93 |
| Error Density | 4 |  |  |  |  |  |  |  |  |  |  |  |
| Seed size | 2 | 0.38 | 14.36^***^ | 11.67^**^ | 6.25^*^ | 4.41^*^ | 8.36^**^ | 1.69 | 7.43^**^ | 5.59^*^ | 3.33 | 4.90^*^ |
| Density*Size | 4 | 2.11 | 0.44 | 0.43 | 1.15 | 1.17 | 1.15 | 0.59 | 0.02 | 0.56 | 0.84 | 0.35 |
| Error | 12 |  |  |  |  |  |  |  |  |  |  |  |

*, P<0.05; **P<0.01; ***, P<0.001.
