# Supplementary material for: The Effects of Seed Size on Hybrids Formed between Oilseed Rape (Brassica napus) and Wild Brown Mustard (B. juncea)
Source: PLoS One. 2012 Jun 22;7(6):e39705. doi: 10.1371/journal.pone.0039705 (PMC3382164; doi:10.1371/journal.pone.0039705)
Supplement: Table S2 — F-values from a split-plot ANOVA on the plant growth characteristics of transgenic hybrid, non- transgenic hybrid, wild mustard and transgenic oilseed rape in the multi-culture experiment. (DOC) [file pone.0039705.s003.doc]

Table S2. F-values from a split-plot ANOVA on the plant growth characteristics of transgenic hybrid, non- transgenic hybrid, wild mustard and transgenic oilseed rape in the multi-culture experiment

|  | df | Emergence rate | Days to flowering | Biomass | Seed number | Seed weight | Reprod. allocation | Thousand-seed weight | % of large seeds | % of medium seeds | % of small seeds |
| --- | --- | --- | --- | --- | --- | --- | --- | --- | --- | --- | --- |
| Block | 2 | 4.07 ^*^ | 1.49 | 1.40 | 0.35 | 0.40 | 0.58 | 1.45 | 1.72 | 0.39 | 0.03 |
| Density (D) | 1 | 52.86 ^*^ | 7.02 | 27.44 ^*^ | 2508 ^**^ | 195.2^*^ | 0.24 | 0.34 | 10.17 | 25.57^*^ | 0.80 |
| Error Density | 2 |  |  |  |  |  |  |  |  |  |  |
| Seed size (S) | 2 | 1.64 | 14.88 ^***^ | 9.46 ^***^ | 4.09 ^*^ | 3.08 ^*^ | 0.03 | 5.20 ^**^ | 0.49 | 0.10 | 0.47 |
| Plant type (P) | 3 | 8.00 ^***^ | 169.9 ^***^ | 50.57 ^***^ | 56.90 ^***^ | 62.79^***^ | 201.9 ^***^ | 147.0 ^***^ | 69.15 ^***^ | 8.47 ^***^ | 41.55 ^***^ |
| D*S | 2 | 0.07 | 0.81 | 0.11 | 0.82 | 0.33 | 0.46 | 0.62 | 2.45 | 0.73 | 0.13 |
| D*P | 3 | 4.62 ^**^ | 5.02 ^**^ | 2.54 | 4.21 ^*^ | 5.16 ^**^ | 2.99 ^*^ | 4.36 ^**^ | 5.92 ^**^ | 0.22 | 1.63 |
| S*P | 6 | 0.61 | 1.54 | 3.66 ^**^ | 3.09 ^*^ | 2.99^*^ | 1.81 | 1.48 | 1.16 | 0.69 | 0.56 |
| D*S*P | 6 | 0.59 | 1.42 | 0.86 | 0.84 | 0.78 | 0.53 | 0.11 | 1.93 | 1.75 | 0.10 |
| Error | 43 |  |  |  |  |  |  |  |  |  |  |

*, P<0.05; **P<0.01; ***, P<0.001.
